# Supplementary material for: Discrete Event Simulation Model for Cost-Effectiveness Evaluation of Screening for Asymptomatic Patients with Lower Extremity Arterial Disease
Source: Int J Environ Res Public Health. 2022 Sep 19;19(18):11792. doi: 10.3390/ijerph191811792 (PMC9517120; doi:10.3390/ijerph191811792)
Supplement: Supplementary file 1 [file ijerph-19-11792-s001.zip › ijerph-1820451-supplementary.pdf]

Supplementary material

Table S1: Description of packages used in R

Name	Maintainer	Brief description	Link
simmer: Discrete-Event Simulation for R	Iñaki Ucar	A process-oriented and trajectory-based Discrete-Event Simulation (DES) package for R.	https://CRAN.R-project.org/package=simmer
Rfast: A Collection of Efficient and Extremely Fast R Functions	Manos Papadakis	A collection of fast (utility) functions for data analysis.	https://CRAN.R-project.org/package=Rfast
Runuran: R Interface to the 'UNU.RAN' Random Variate Generators	Josef Leydold	Interface to the 'UNU.RAN' library for Universal Non-Uniform RANdom variate generators.	https://CRAN.R-project.org/package=Runuran
extraDistr: Additional Univariate and Multivariate Distributions	Tymoteusz Wolodzko	Density, distribution function, quantile function and random generation for a number of univariate and multivariate distributions.	https://CRAN.R-project.org/package=extraDistr
EnvStats: Package for Environmental Statistics, Including US EPA Guidance	Alexander Kowarik	Graphical and statistical analyses of environmental data, with focus on analyzing chemical concentrations and physical parameters, usually in the context of mandated environmental monitoring.	https://CRAN.R-project.org/package=EnvStats
Rcpp: Seamless R and C++ Integration	Dirk Eddelbuettel	The 'Rcpp' package provides R functions as well as C++ classes which offer a seamless integration of R and C++.	https://CRAN.R-project.org/package=Rcpp
dplyr: A Grammar of Data Manipulation	Hadley Wickham	A fast, consistent tool for working with data frame like objects, both in memory and out of memory.	https://CRAN.R-project.org/package=dplyr
purrr: Functional Programming Tools	Lionel Henry	A complete and consistent functional programming toolkit for R.	https://CRAN.R-project.org/package=purrr

stringr: Simple, Consistent Wrappers for Common String Operations	Hadley Wickham	A consistent, simple and easy to use set of wrappers around the fantastic 'stringi' package.	https://CRAN.R-project.org/package=stringr;
svMisc: 'SciViews' - Miscellaneous Functions	Philippe Grosjean	Miscellaneous functions for 'SciViews' or general use: manage a temporary environment attached to the search path for temporary variables you do not want to save() or load().	https://CRAN.R-project.org/package=svMisc
ggpubr: 'ggplot2' Based Publication Ready Plots	Alboukadel Kassambara	'ggpubr' provides some easy-to-use functions for creating and customizing 'ggplot2'- based publication ready plots.	https://CRAN.R-project.org/package=ggpubr
survival: Survival Analysis	Terry M Therneau	Contains the core survival analysis routines, including definition of Surv objects, Kaplan-Meier and Aalen-Johansen (multi-state) curves, Cox models, and parametric accelerated failure time models.	https://CRAN.R-project.org/package=survival
survminer: Drawing Survival Curves using 'ggplot2'	Alboukadel Kassambara	Contains the function 'ggsurvplot()' for drawing easily beautiful and 'ready-to-publish' survival curves with the 'number at risk' table and 'censoring count plot'.	https://CRAN.R-project.org/package=survminer

Table S2: Parameters for modeling the time of occurrence of simulated events

Parameters	Probability distribution	Values	Sensitivity analysis	Source
Occurrence of IC	Exponential	rate = 0.021	Exponential parameter varied by ± 20% with uniform distribution	[1]
Occurrence of CLI (PAD to CLI)	exponential	rate = 0.004	Exponential parameter varied by ± 20% with uniform distribution	[1]
Occurrence of CLI (IC to CLI)	exponential	rate = 0.064	Exponential parameter varied by ± 20% with uniform distribution	[1]
Occurrence of amputation (Fontaine IIa, IIb)	Weibull	shape=0.40, scale=2010		[2]
Occurrence of amputation (Fontaine III, IV)	Weibull	shape=0.46, scale=73.44	Varying only scale parameter	[2]
Time to death (female)	Weibull	shape=3.52, scale=31.98	Variation of the scale parameter; uniform distribution within ± 20% interval	[3]
Time to death (male)	Weibull	shape=2.64, scale=36.84		[3]
Death after amputation	Weibull	shape=0.61, scale=5.48		[4]
		Vessel patency		
Primary PTA (IC)	Weibull	shape = 0.87 scale= 28.13	Variation of the scale parameter; uniform distribution within ± 20% interval	[5–8]
Primary PTA (CLI)	Weibull	shape = 0.93 scale = 10.17		
Primary PTA/S (IC)	Weibull	shape = 0.80 scale = 28.19		
Primary PTA/S (CLI)	Weibull	shape = 0.88 scale = 9.37		
Primary (autologous; IC)	Weibull	shape = 1.04 scale = 31.37		
Primary (autologous ;CLI)	Weibull	shape = 1.16 scale = 11.66		
Primary (graft; IC)	Weibull	shape = 1.22 scale = 20.78		
Primary (graft; CLI)	Weibull	shape = 1.42 scale = 9.01		
Secondary PTA (IC)	Weibull	shape = 0.79 scale = 67.10		
Secondary PTA (CLI)	Weibull	shape = 0.82 scale = 21.74		
Secondary PTA/S (IC)	Weibull	shape = 0.83 scale = 47.21		
Secondary PTA/S (CLI)	Weibull	shape = 0.87 scale = 16.09		
Secondary (autologous; IC)	Weibull	shape = 0.96 scale = 56.49		
Secondary (autologous; CLI)	Weibull	shape = 1.01 scale = 21.96		
Sekundární (graft; IC)	Weibull	shape = 0.97 scale = 33.00		
Sekundární (graft; CLI)	Weibull	shape = 1.21 scale = 14.38		
		Time to next examination		

Time to next preventive examination	Uniform	Min=1,8<br>Max= 2,2	Fixed	[9]
Time of effect exercise therapy	Triangular	Min= 1,5<br>Mode= 2<br>Max = 2,5	Fixed	
Time between followed diagnostic	-	1 year	Uniform distribution (min = 0.8, max = 1.2)	

Primary and secondary patency for endovascular interventions were simulated based on a study Vossen et al. [5]. No statistically significant effect of Fontaine classification was found in the study for primary patency. A significant effect was found for secondary patency, but the value of the effect was not reported in the study. In other studies, the effect of CLI on primary patency was reported, e.g., Nishibe et al. [6] reports an HR of 2.5 (95% CI 1.08-5.83). Probability distribution values for primary patency obtained from the analysis of published Kaplan–Meier curves from the study of Vossen et al. [5] were adjusted based on simulations, so that an HR between IC and CLI was required to be 2.5. An overview of the probability distribution settings simulated for IC and CLI based on the data of the study by Vossen et al. [5] can be seen in Table S1.

Primary bypass patency was derived from PTA data. A study by Antoniou et al. [7] (comparison of bypass and PTA) shows an odds ratio of 1.44 for IC and 0.95 for CLI (for 4-year primary patency). The OR values were converted to RR, and therefore a value of 1.11 is considered for IC and a value of 0.98 for CLI. The difference of autologous and artificial bypasses was simulated based on Cochrane's systematic search by Ambler and Twine [8]. The size of the effect was based on the data from a comparison of 5-year primary patency, and the OR of 0.47 was recalculated to an RR of 0.62. In the Ambler and Twine study [8], the data were not published separately for IC and CLI, and therefore the considered RR is the same for IC and CLI.

The same process was used for secondary throughput data. As a suitable study evaluating the effect of CLI on secondary patency versus IC was not found, the same magnitude of effect as reported by Nishibe et al. [6] for primary patency was used (HR 2.5 (95% CI 1.08-5.83)). The secondary bypass patency was again derived from PTA data. In a study by Antoniou et al. [7], an OR 0.9 (for 4-year secondary patency) without distinguishing between area of intervention and disease severity (IC vs. CLI) was used. The OR values were converted to RR, and the considered value was 0.97. Input parameters for autologous and artificial bypasses were again based on Cochrane's systematic search by Ambler and Twine [8]. The size of the effect was based on comparison of 5-year secondary patency and the OR 0.41 was recalculated to 0.59 RR.

Table S3: Parameters and assumptions used in the model

Parameters	Value	Sensitivity analysis	Source
Time to death with PAD	RR = 1,6 relative to úmrtí	Fixed	[10]
Time to death with IC	RR = 3,1 relative to úmrtí	Fixed	[11]
Time to death with CLI	RR = 2,0 relative to IC	Fixed	[12]
Effect of pharmacological treatment	HR = 0,75	Fixed	[13,14]
Vein for bypass	60 % autologous	beta(39.4, 26.27)	
Preventive examination	65 % regularly<br>25 % irregularly<br>10 % do not attend	Fixed	[15]
Irregular check-ups – prob. of attending	50 %	beta(12, 12)	
Fontaine class distribution in occurrence of IC	IIa: 66,8 %<br>IIb: 33,2 %	IIa: beta (32.53, 16.17)	[16]
Fontaine class distribution in occurrence oCLI	III 66,7 %<br>IV 33,3 %	III: beta (32.63, 16.29)	[16]
Exercise effect	21,8 % worsening<br>36,7 % unchanged<br>41,6 % improvement	worsening: beta (77.98, 280)<br>improvement: beta (57.98, 81.40)	[17]
Efekt bez cvičení	19.4 % worsening<br>48.1 % unchanged<br>32.5 % improvement	worsening: beta(80.41, 334)<br>improvement: beta(67.18, 140)	[17]
Type of amputation	BKA = 68,3 %<br>AKA = 31,7 %	BKA: beta(31.02, 14.40)	[18]
30-day mortality PTA	IC = 0,2 %<br>CLI = 2,8 %	IC: beta(99.80, 49799)<br>CLI: beta(97.17, 3373)	[18]<br>[19]
30-day mortality PTA/S	IC = 0,4 %<br>CLI = 2,8 %	IC: beta(99.60, 24799)<br>CLI: beta(97.17, 3373)	[20]<br>[19]
30-day mortality bypass	IC = 0,7 %<br>CLI = 3,3 %	IC: beta(99.29, 14085)<br>CLI: beta(96.67, 2833)	[20]<br>[19]
30-day mortality amputation	BKA = 8,9 %<br>AKA = 27,7 %	BKA: beta(91.01, 932)<br>AKA: beta(72.02, 188)	[4]<br>[4]
30-day morbidity PTA	IC = 3,8 %<br>CLI = 35,8 %	IC: beta(96.16, 2434)<br>CLI: beta(63.84, 114.49)	[7]
30-day morbidity PTA/S	IC = 2,5 %<br>CLI = 35,8 %	IC: beta(97.48, 3802)<br>CLI: beta(63.84, 114.49)	[20]<br>[7]
30-day morbidity bypass	IC = 9,5 %<br>CLI = 46,3 %	IC: beta(90.41, 861)<br>CLI: beta(53.24, 61.75)	[7]
Distribution of complication for bypass according DRG	Mild:qa 43 %<br>Severe: 57 %	CC=2-3: beta(56.57, 74.99)	[21]
Reoperation possible	IC = 95 %<br>CLI = 98,45 %	IC: beta(4.05, 0.21)<br>CLI: beta(0.57, 0.009)	[18,22]
DM	Male 13,34 %<br>Female 9,84 %	Male: beta(86.53, 562.10)<br>Female: beta(90.06, 825.20)	[23]
Hypertension	Male 21,89 %<br>Female 22,26 %	Male: beta(183.42, 23.13)<br>Female: beta(77.52, 270.72)	[24]
Dyslipidemia	88,8 %	beta(10.31, 1.30)	[23]
Smoking status	Male 35,2 %<br>Female 27,2 %	Male: beta(64.45, 118.64)<br>Female: beta(72.53, 194.12)	[25]
Technical success PTA	86.5 %	beta(15.16, 2.89)	[26]
Technical success PTA/S	95 %	beta(10.38, 1.69)	[27]
Technical success Bypass	100 %	Fixed	[28]
Amputation in CVSP	Yes = 46,32 %	beta(53.22, 61.67)	[10]

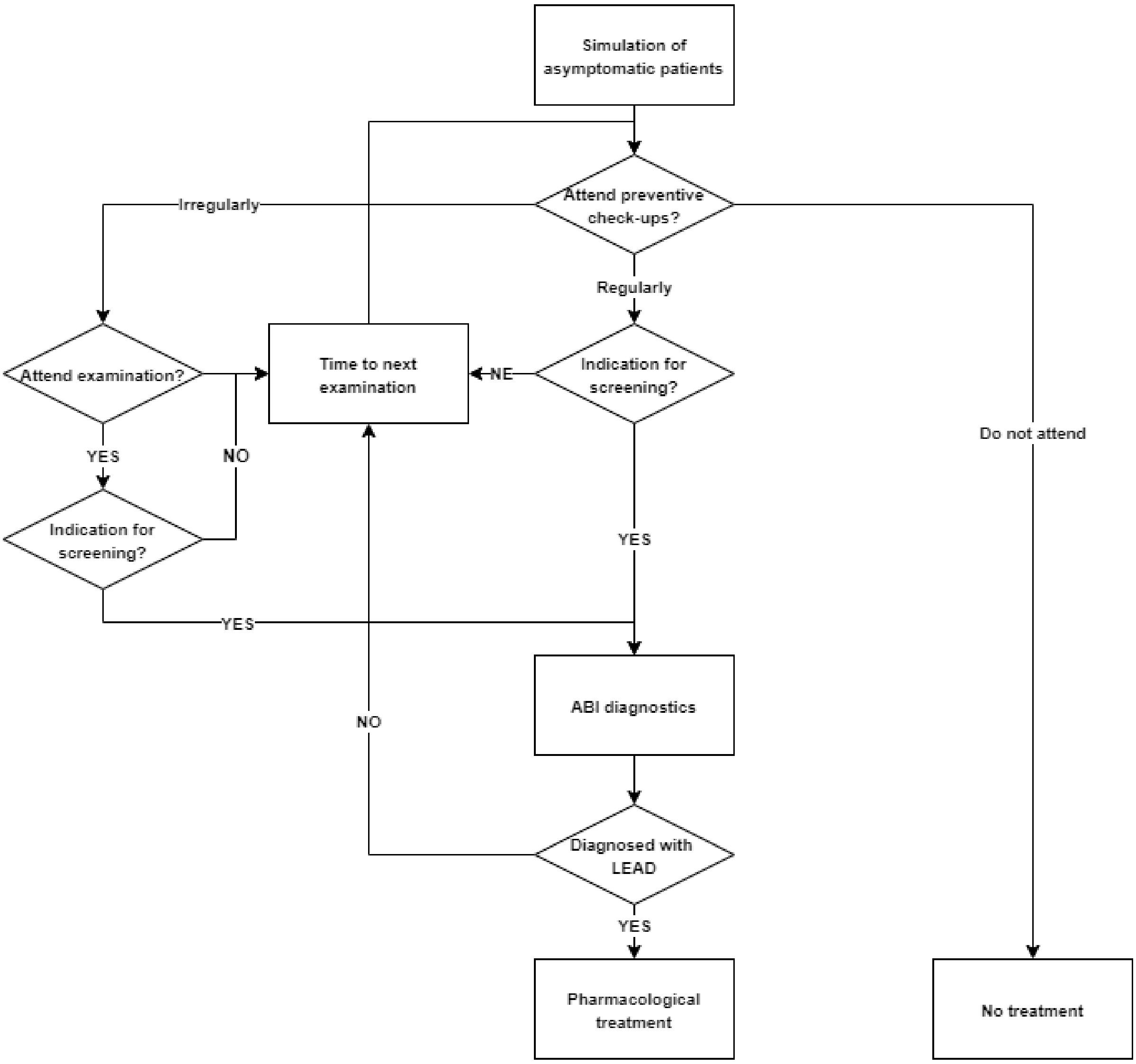

Figure S1: Model logic diagram – screening with ABI

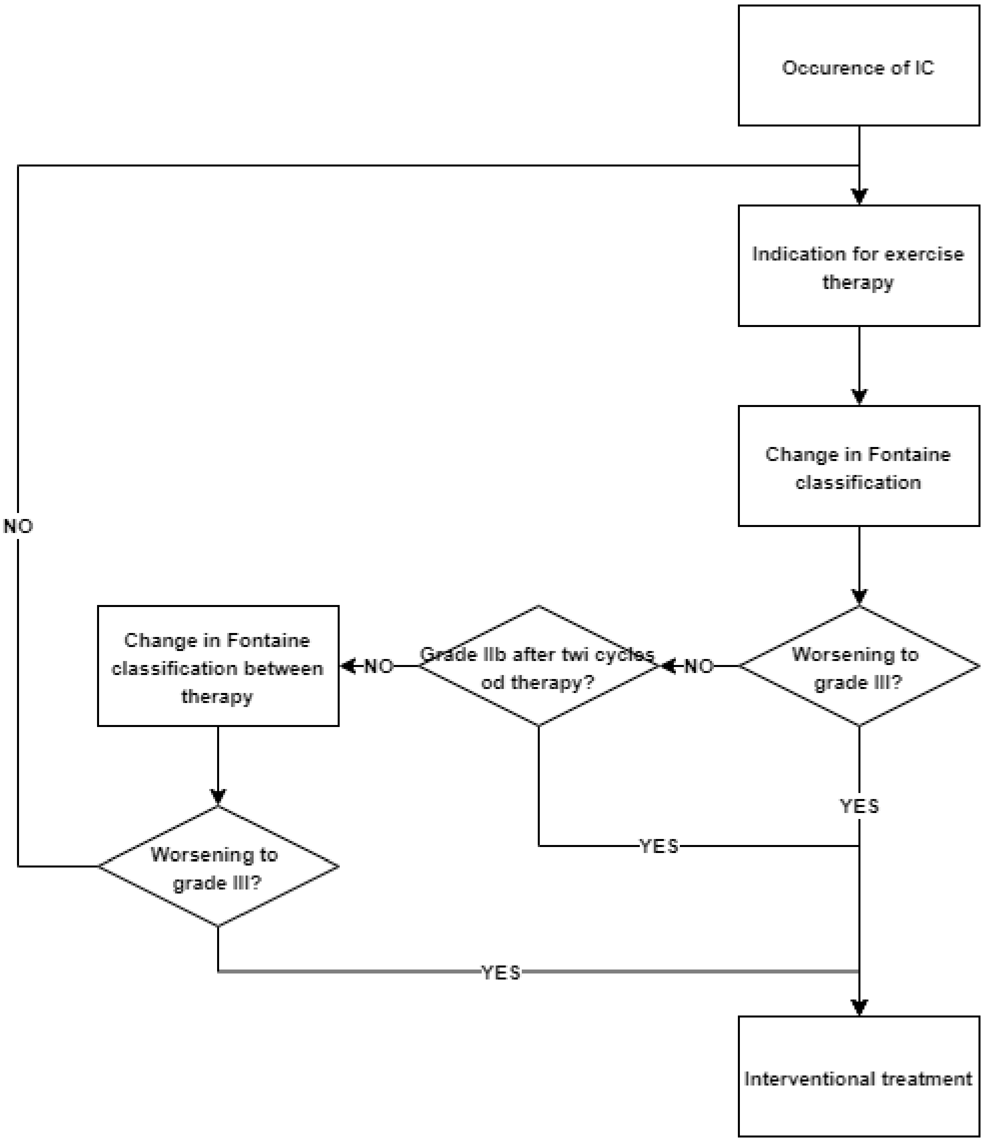

Figure S2: Model logic diagram – Exercise therapy for Fontaine classification IIa and IIb

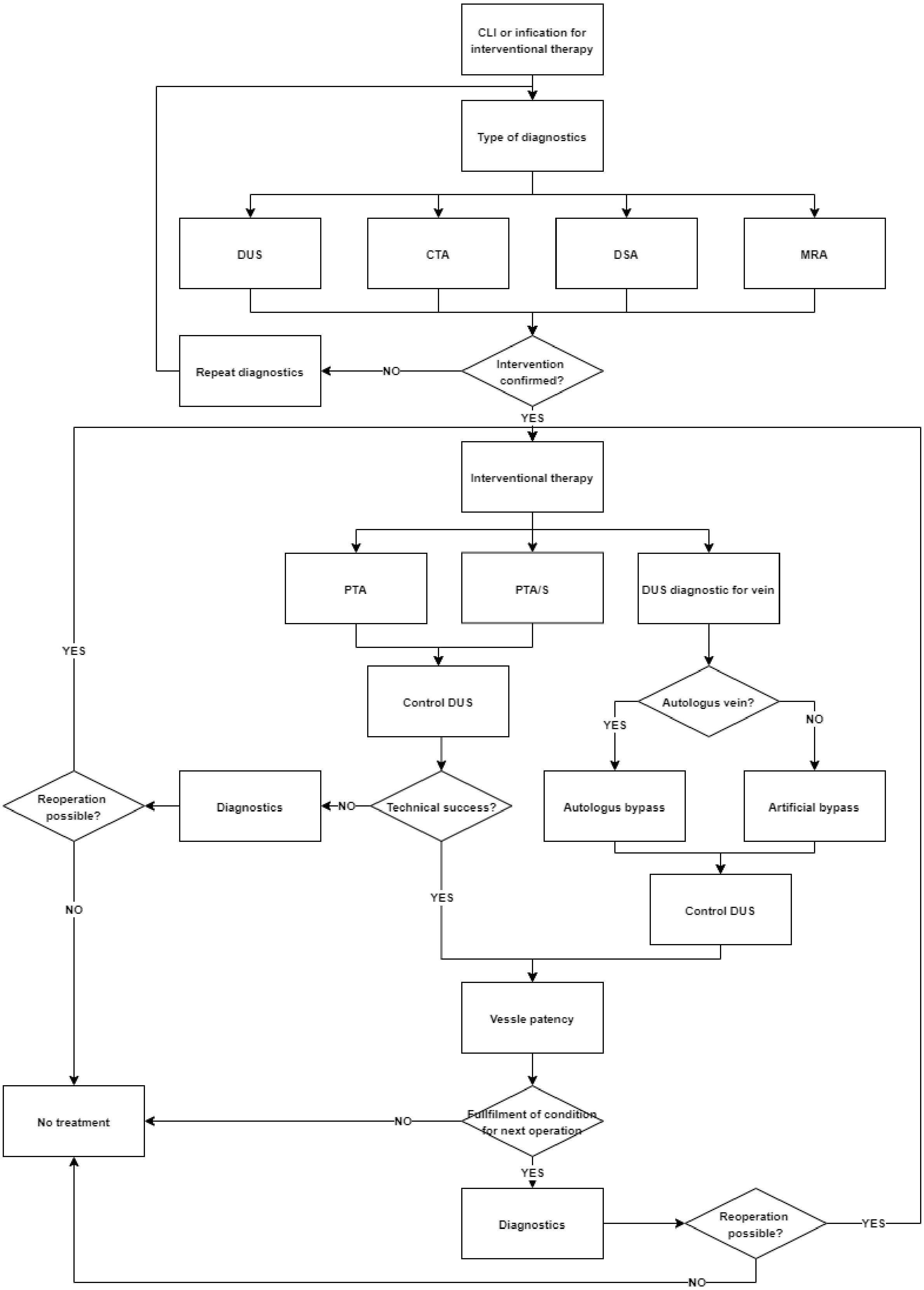

Figure S3: Model logic diagram – Interventional therapy decision

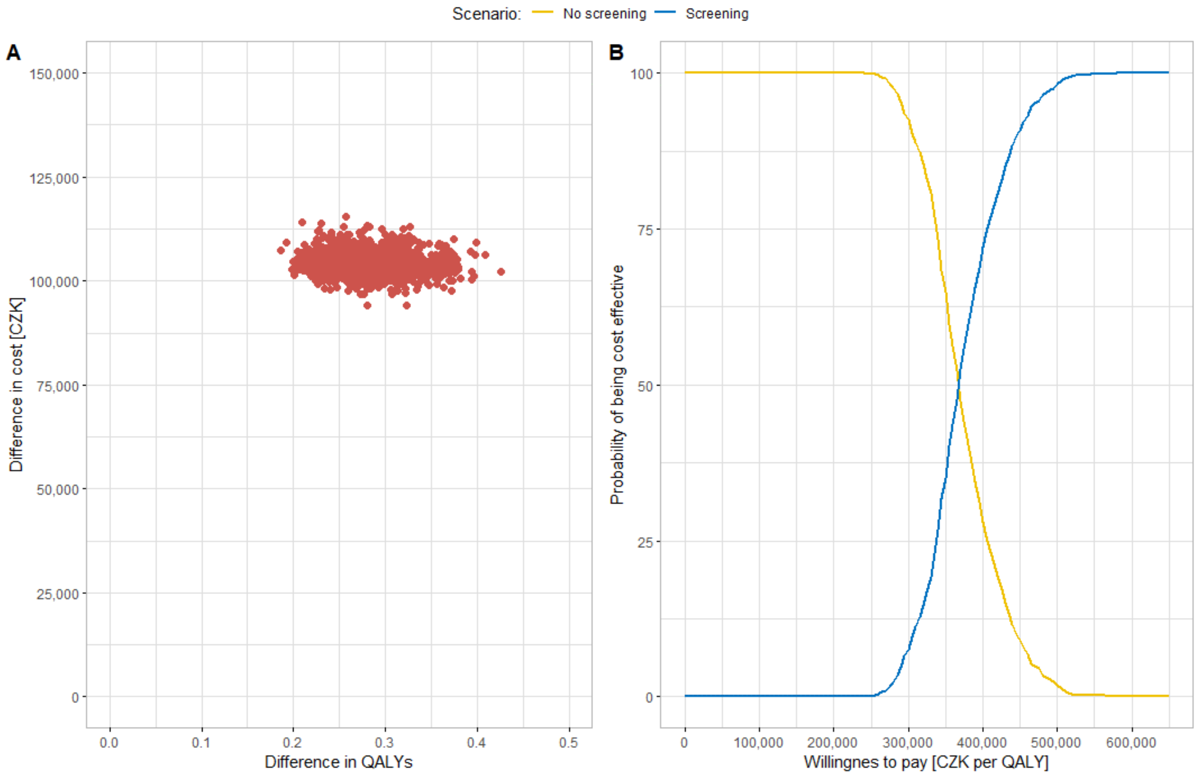

Figure S4: Cost-effectiveness scatter plane using different pseudo-random numbers; **B:** Cost-effectiveness acceptability curve for probabilistic analysis using different pseudo-random numbers.

The cost-effectiveness scatter plane in Figure S1 in Part A shows the resulting ICER values obtained using different pseudorandom numbers for each iteration. All results (red dots in figure S4) are again in the upper right quadrant, where screening is a more costly, while more effective, intervention. The ICER values were below the WTP for all iterations in this case as well, and therefore screening is a cost-effective intervention in all simulation iterations. The average mean value of the cost difference and the effect difference at a discount rate of 3% was CZK 104,304 and 0.29, respectively, and the resulting ICER value was CZK 363,642 per QALY. In part B Figure S1, it can be seen that from a WTP greater than CZK 550,000, that the ICER of all iterations are cost-effective.

References

1. Ezeofor, V.S.; Bray, N.; Bryning, L.; Hashmi, F.; Hoel, H.; Parker, D.; Edwards, R.T. Economic Model to Examine the Cost-Effectiveness of FlowOx Home Therapy Compared to Standard Care in Patients with Peripheral Artery Disease. *PLoS ONE* **2021**, *16*, e0244851. https://doi.org/10.1371/journal.pone.0244851.
2. Moussa Pacha, H.; Mallipeddi, V.P.; Afzal, N.; Moon, S.; Kaggal, V.C.; Kalra, M.; Oderich, G.S.; Wennberg, P.W.; Rooke, T.W.; Scott, C.G.; et al. Association of Ankle-Brachial Indices With Limb Revascularization or Amputation in Patients With Peripheral Artery Disease. *JAMA Netw Open* **2018**, *1*, e185547. https://doi.org/10.1001/jamanetworkopen.2018.5547.
3. Úmrtnostní Tabulky za ČR, Regiony Soudržnosti a kraje. 2019–2020. Available online: https://www.czso.cz/csu/czso/umrtnostni-tabulky-za-cr-regiony-soudrznosti-a-kraje-i09aftm7w4 (accessed on 14 February 2022).
4. Abry, L.; Weiss, S.; Makaloski, V.; Haynes, A.G.; Schmidli, J.; Wyss, T.R. Peripheral Artery Disease Leading to Major Amputation: Trends in Revascularization and Mortality Over 18 Years. *Ann. Vasc. Surg.* **2022**, *78*, 295–301. https://doi.org/10.1016/j.avsg.2021.04.037.
5. Vossen, R.J.; Vahl, A.C.; Leijdekkers, V.J.; Montauban van Swijndregt, A.D.; Balm, R. Long-Term Clinical Outcomes of Percutaneous Transluminal Angioplasty with Optional Stenting in Patients with Superficial Femoral Artery Disease: A Retrospective, Observational Analysis. *Eur. J. Vasc. Endovasc. Surg.* **2018**, *56*, 690–698. https://doi.org/10.1016/j.ejvs.2018.06.063.
6. Nishibe, T.; Yamamoto, K.; Seike, Y.; Ogino, H.; Nishibe, M.; Koizumi, J.; Dardik, A. Endovascular Therapy for Femoropopliteal Artery Disease and Association of Risk Factors With Primary Patency: The Implication

of Critical Limb Ischemia and TASC II C/D Disease. *Vasc Endovasc. Surg* **2015**, *49*, 236–241. https://doi.org/10.1177/1538574415614406.
7. Antoniou, G.A.; Georgiadis, G.S.; Antoniou, S.A.; Makar, R.R.; Smout, J.D.; Torella, F. Bypass Surgery for Chronic Lower Limb Ischaemia. *Cochrane Database Syst. Rev.* **2017**, *2017*, CD002000. https://doi.org/10.1002/14651858.CD002000.pub3.
8. Ambler, G.K.; Twine, C.P. Graft Type for Femoro-Popliteal Bypass Surgery. *Cochrane Database Syst. Rev.* **2018**, *2018*, CD001487. https://doi.org/10.1002/14651858.CD001487.pub3.
9. Lane, R.; Harwood, A.; Watson, L.; Leng, G.C. Exercise for Intermittent Claudication. *Cochrane Database Syst. Rev.* **2017**, *2017*, CD000990. https://doi.org/10.1002/14651858.CD000990.pub4.
10. Sartipy, F.; Sigvant, B.; Lundin, F.; Wahlberg, E. Ten Year Mortality in Different Peripheral Arterial Disease Stages: A Population Based Observational Study on Outcome. *Eur. J. Vasc. Endovasc. Surg.* **2018**, *55*, 529–536. https://doi.org/10.1016/j.ejvs.2018.01.019.
11. Criqui, M.H.; Langer, R.D.; Fronek, A.; Feigelson, H.S.; Klauber, M.R.; McCann, T.J.; Browner, D. Mortality over a Period of 10 Years in Patients with Peripheral Arterial Disease. *N. Engl. J. Med.* **1992**, *326*, 381–386. https://doi.org/10.1056/NEJM199202063260605.
12. Norgren, L.; Hiatt, W.R.; Dormandy, J.A.; Nehler, M.R.; Harris, K.A.; Fowkes, F.G.R. Inter-Society Consensus for the Management of Peripheral Arterial Disease (TASC II). *J. Vasc. Surg.* **2007**, *45*, S5–S67. https://doi.org/10.1016/j.jvs.2006.12.037.
13. Golledge, J.; Drovandi, A. Evidence-Based Recommendations for Medical Management of Peripheral Artery Disease. *JAT* **2021**, *28*, 573–583. https://doi.org/10.5551/jat.62778.
14. Bevan, G.H.; White Solaru, K.T. Evidence-Based Medical Management of Peripheral Artery Disease. *Arterioscler. Thromb. Vasc. Biol.* **2020**, *40*, 541–553. https://doi.org/10.1161/ATVBAHA.119.312142.
15. *Čechům se Během Pandemie Zhoršil Zdravotní Stav, Odkládali Návštěvu Lékaře*; Ministerstvo Zdravotnictví: Prague, Czech Republic.
16. Vaidya, A.; Kleinegris, M.-C.; Severens, J.L.; Ramaekers, B.L.; ten Cate-Hoek, A.J.; ten Cate, H.; Joore, M.A. Comparison of EQ-5D and SF-36 in Untreated Patients with Symptoms of Intermittent Claudication. *J. Comp. Eff. Res.* **2018**, *7*, 535–548. https://doi.org/10.2217/cer-2017-0029.
17. McDermott, M.M.; Tian, L.; Criqui, M.H.; Ferrucci, L.; Greenland, P.; Guralnik, J.M.; Kibbe, M.R.; Li, L.; Sufit, R.; Zhao, L.; et al. Perceived Versus Objective Change in Walking Ability in Peripheral Artery Disease: Results from 3 Randomized Clinical Trials of Exercise Therapy. *JAHA* **2021**, *10*, e017609. https://doi.org/10.1161/JAHA.120.017609.
18. Simpson, E.L.; Kearns, B.; Stevenson, M.D.; Cantrell, A.J.; Littlewood, C.; Michaels, J.A. Enhancements to Angioplasty for Peripheral Arterial Occlusive Disease: Systematic Review, Cost-Effectiveness Assessment and Expected Value of Information Analysis. *Health Technol. Assess.* **2014**, *18*, 1–252. https://doi.org/10.3310/hta18100.
19. Darling, J.D.; McCallum, J.C.; Soden, P.A.; Korepta, L.; Guzman, R.J.; Wyers, M.C.; Hamdan, A.D.; Schermerhorn, M.L. Results for Primary Bypass versus Primary Angioplasty/Stent for Lower Extremity Chronic Limb-Threatening Ischemia. *J. Vasc. Surg.* **2017**, *66*, 466–475. https://doi.org/10.1016/j.jvs.2017.01.024.
20. Liang, P.; Li, C.; O'Donnell, T.F.X.; Lo, R.C.; Soden, P.A.; Swerdlow, N.J.; Schermerhorn, M.L. In-Hospital versus Postdischarge Major Adverse Events within 30 Days Following Lower Extremity Revascularization. *J. Vasc. Surg.* **2019**, *69*, 482–489. https://doi.org/10.1016/j.jvs.2018.06.207.
21. *Stažení Distribučního Balíčku CZ-DRG v4 Revize 1*; Ministerstvo Zdravotnictví: Prague, Czech Republic, 2021.
22. de Vries, S.O.; Visser, K.; de Vries, J.A.; Wong, J.B.; Donaldson, M.C.; Hunink, M.G.M. Intermittent Claudication: Cost-Effectiveness of Revascularization versus Exercise Therapy. *Radiology* **2002**, *222*, 25–36. https://doi.org/10.1148/radiol.2221001743.
23. Cífková, R.; Bruthans, J.; Adámková, V.; Jozífová, M.; Galovcová, M.; Wohlfahrt, P.; Krajčoviechová, A.; Petržílková, Z.; Lánská, V.; Poledne, R.; et al. The prevalence of major cardiovascular risk factors in the Czech population in 2006–2009. The Czech post-MONICA study. *Cor Vasa* **2011**, *53*, 220–229. https://doi.org/10.33678/cor.2011.050.
24. van Rossum, C.T.M.; van de Mheen, H.; Witteman, J.C.M.; Hofman, A.; Mackenbach, J.P.; Grobbee, D.E. Prevalence, Treatment, and Control of Hypertension by Sociodemographic Factors Among the Dutch Elderly. *Hypertension* **2000**, *35*, 814–821. https://doi.org/10.1161/01.HYP.35.3.814.
25. Csémy, L.; Dvo, Z.; Fialová, A.; Kodl, M.; Malý, M.; Skývová, M. *NARODNI VYZKUM UZIVANI TABAKU A ALKOHOLU V CESKE REPUBLICE 2020*; NAUTA: Kanice, Czech Republic, 2020; 63.
26. Schulte, K.-L.; Hardung, D.; Tiefenbacher, C.; Weiss, T.; Hoffmann, U.; Amendt, K.; Tepe, G.; Heuser, L.; Treszl, A.; Lau, H.-J.; et al. Real-World Outcomes of Endovascular Treatment in a Non-Selected Population with Peripheral Artery Disease—Prospective Study with 2-Year Follow-Up. *Vasa* **2019**, *48*, 433–441. https://doi.org/10.1024/0301-1526/a000798.
27. Saxon, R.R.; Dake, M.D.; Volgelzang, R.L.; Katzen, B.T.; Becker, G.J. Randomized, Multicenter Study Comparing Expanded Polytetrafluoroethylene–Covered Endoprosthesis Placement with Percutaneous

Transluminal Angioplasty in the Treatment of Superficial Femoral Artery Occlusive Disease. *J. Vasc. Interv. Radiol.* **2008**, *19*, 823–832. https://doi.org/10.1016/j.jvir.2008.02.008.
28. van der Zaag, E.S.; Legemate, D.A.; Prins, M.H.; Reekers, J.A.; Jacobs, M.J. Angioplasty or Bypass for Superficial Femoral Artery Disease? A Randomised Controlled Trial. *Eur. J. Vasc. Endovasc. Surg.* **2004**, *28*, 132–137. https://doi.org/10.1016/j.ejvs.2004.04.003.
29. Criqui, M.H.; Aboyans, V. Epidemiology of Peripheral Artery Disease. *Circ. Res.* **2015**, *116*, 1509–1526. https://doi.org/10.1161/CIRCRESAHA.116.303849.
30. Fowkes, F.G.R.; Aboyans, V.; Fowkes, F.J.I.; McDermott, M.M.; Sampson, U.K.A.; Criqui, M.H. Peripheral Artery Disease: Epidemiology and Global Perspectives. *Nat. Rev. Cardiol.* **2017**, *14*, 156–170. https://doi.org/10.1038/nrcardio.2016.179.
31. Criqui, M.H.; Matsushita, K.; Aboyans, V.; Hess, C.N.; Hicks, C.W.; Kwan, T.W.; McDermott, M.M.; Misra, S.; Ujueta, F.; on behalf of the American Heart Association Council on Epidemiology and Prevention; et al. Lower Extremity Peripheral Artery Disease: Contemporary Epidemiology, Management Gaps, and Future Directions: A Scientific Statement From the American Heart Association. *Circulation* **2021**, *144*, e171–e191. https://doi.org/10.1161/CIR.0000000000001005.
32. Thiruvoipati, T. Peripheral Artery Disease in Patients with Diabetes: Epidemiology, Mechanisms, and Outcomes. *WJD* **2015**, *6*, 961. https://doi.org/10.4239/wjd.v6.i7.961.
33. Gerhard-Herman, M.D.; Gornik, H.L.; Barrett, C.; Barshes, N.R.; Corriere, M.A.; Drachman, D.E.; Fleisher, L.A.; Fowkes, F.G.R.; Hamburg, N.M.; Kinlay, S.; et al. 2016 AHA/ACC Guideline on the Management of Patients With Lower Extremity Peripheral Artery Disease: Executive Summary: A Report of the American College of Cardiology/American Heart Association Task Force on Clinical Practice Guidelines. *Circulation* **2017**, *135*, e686–e725. https://doi.org/10.1161/CIR.0000000000000470.
34. Diehm, C.; Allenberg, J.R.; Pittrow, D.; Mahn, M.; Tepohl, G.; Haberl, R.L.; Darius, H.; Burghaus, I.; Trampisch, H.J. Mortality and Vascular Morbidity in Older Adults With Asymptomatic Versus Symptomatic Peripheral Artery Disease. *Circulation* **2009**, *120*, 2053–2061. https://doi.org/10.1161/CIRCULATIONAHA.109.865600.
35. Aboyans, V.; Ricco, J.-B.; Bartelink, M.-L.E.L.; Björck, M.; Brodmann, M.; Cohnert, T.; Collet, J.-P.; Czerny, M.; De Carlo, M.; Debus, S.; et al. 2017 ESC Guidelines on the Diagnosis and Treatment of Peripheral Arterial Diseases, in Collaboration with the European Society for Vascular Surgery (ESVS). *Eur. Heart J.* **2018**, *39*, 763–816. https://doi.org/10.1093/eurheartj/ehx095.
36. McDermott, M.M.; Applegate, W.B.; Bonds, D.E.; Buford, T.W.; Church, T.; Espeland, M.A.; Gill, T.M.; Guralnik, J.M.; Haskell, W.; Lovato, L.C.; et al. Ankle Brachial Index Values, Leg Symptoms, and Functional Performance Among Community-Dwelling Older Men and Women in the Lifestyle Interventions and Independence for Elders Study. *JAHA* **2013**, *2*, e000257. https://doi.org/10.1161/JAHA.113.000257.
37. McDermott, M.M.; Mehta, S.; Liu, K.; Guralnik, J.M.; Martin, G.J.; Criqui, M.H.; Greenland, P. Leg Symptoms, the Ankle-Brachial Index, and Walking Ability in Patients with Peripheral Arterial Disease. *J. Gen. Intern. Med.* **1999**, *14*, 173–181. https://doi.org/10.1046/j.1525-1497.1999.00309.x.
38. McDermott, M.M.; Liu, K.; Greenland, P.; Guralnik, J.M.; Criqui, M.H.; Chan, C.; Pearce, W.H.; Schneider, J.R.; Ferrucci, L.; Celic, L.; et al. Functional Decline in Peripheral Arterial Disease: Associations With the Ankle Brachial Index and Leg Symptoms. *JAMA* **2004**, *292*, 453. https://doi.org/10.1001/jama.292.4.453.
39. Fontaine, R.; Kim, M.; Kieny, R. Surgical Treatment of Peripheral Circulation Disorders. *Helv Chir Acta* **1954**, *21*, 499–533.
40. Karnon, J.; Stahl, J.; Brennan, A.; Caro, J.J.; Mar, J.; Möller, J. Modeling Using Discrete Event Simulation: A Report of the ISPOR-SMDM Modeling Good Research Practices Task Force-4. *Value Health* **2012**, *15*, 821–827. https://doi.org/10.1016/j.jval.2012.04.013.
41. R Core Team. *R: A Language and Environment for Statistical Computing*; R Core Team: Vienna, Austria, 2021.
42. ČSÚ—Věková Struktura. Available online: https://www.czso.cz/staticke/animgraf/cz/index.html?lang=cz (accessed on 18 June 2022).
43. Pollak, A.W.; Norton, P.T.; Kramer, C.M. Multimodality Imaging of Lower Extremity Peripheral Arterial Disease: Current Role and Future Directions. *Circ. Cardiovasc. Imaging* **2012**, *5*, 797–807. https://doi.org/10.1161/CIRCIMAGING.111.970814.
44. Janssen, M.F.; Szende, A.; Cabases, J.; Ramos-Goñi, J.M.; Vilagut, G.; König, H.H. Population Norms for the EQ-5D-3L: A Cross-Country Analysis of Population Surveys for 20 Countries. *Eur. J. Health Econ.* **2019**, *20*, 205–216. https://doi.org/10.1007/s10198-018-0955-5.
45. de Vries, M.; Ouwendijk, R.; Kessels, A.G.; de Haan, M.W.; Flobbe, K.; Hunink, M.G.M.; van Engelshoven, J.M.A.; Nelemans, P.J. Comparison of Generic and Disease-Specific Questionnaires for the Assessment of Quality of Life in Patients with Peripheral Arterial Disease. *J. Vasc. Surg.* **2005**, *41*, 261–268. https://doi.org/10.1016/j.jvs.2004.11.022.
46. Sculpher, M.; Michaels, J.; Mckenna, M.; Minor, J. A Cost-Utility Analysis of Laser-Assisted Angioplasty for Peripheral Arterial Occlusions. *Int. J. Technol. Assess. Health Care* **1996**, *12*, 104–125. https://doi.org/10.1017/S0266462300009430.

47. Státní Ústav pro Kontrolu Léčiv. Available online: https://www.sukl.cz/modules/medication/search.php (accessed on 29 May 2022).
48. info@aion.cz, A.C.-428/2020 Sb. Vyhláška o Stanovení Hodnot Bodu, Výše Úhrad Hrazených Služeb a Regulačních Omezení pro Rok. 2021. Available online: https://www.zakonyprolidi.cz/cs/2020-428 (accessed on 29 May 2022).
49. Klasnová, N. Ekonomická Náročnost Protetické a Terapeutické Péče Dolní Končetiny Po Transfemurální Amputaci. Master's Thesis, ČVUT, Fakulta Biomedicínského Inženýrství v Kladně, Kladno, Czech Republic, 2019.
50. Půlpán, R. Úvodní Slovo. *Ortop. Protet. Odb. Časopis Fed. Ortop. Protetiků Tech. Oborů* **2018**.
51. *Ústav Zdravotnických Informací a Statistiky Zdravotnická Ročenka 2018*; České Republiky: Prague, Czech Republic, 2018.
52. Wilkins, E.; Wilson, L.; Wickramasinghe, K.; Bhatnagar, P.; Leal, J.; Luengo-Fernandez, R.; Burns, R.; Rayner, M.; Townsend, N. *European Cardiovascular Disease Statistics 2017*; European Heart Network: Brusel, Brussels, 2017.
53. Briggs, A.H.; Weinstein, M.C.; Fenwick, E.A.L.; Karnon, J.; Sculpher, M.J.; Paltiel, A.D. Model Parameter Estimation and Uncertainty Analysis: A Report of the ISPOR-SMDM Modeling Good Research Practices Task Force Working Group–6. *Med Decis Mak.* **2012**, *32*, 722–732. https://doi.org/10.1177/0272989X12458348.
54. Vaidya, A.; Joore, M.A.; ten Cate-Hoek, A.J.; ten Cate, H.; Severens, J.L. Screen or Not to Screen for Peripheral Arterial Disease: Guidance from a Decision Model. *BMC Public Health* **2014**, *14*, 89. https://doi.org/10.1186/1471-2458-14-89.
55. Hirsch, A.T. Peripheral Arterial Disease Detection, Awareness, and Treatment in Primary Care. *JAMA* **2001**, *286*, 1317. https://doi.org/10.1001/jama.286.11.1317.
56. Sutkowska, E.E.; Sokołowski, M.; Zdrojowy, K.; Dragan, S. Active Screening for Diabetic Foot—Assessment of Health Care Professionals' Compliance to It. *Clin. Diabetol.* **2016**, *5*, 83–87. https://doi.org/10.5603/DK.2016.0014.
57. Aboyans, V.; Criqui, M.H.; Abraham, P.; Allison, M.A.; Creager, M.A.; Diehm, C.; Fowkes, F.G.R.; Hiatt, W.R.; Jönsson, B.; Lacroix, P.; et al. Measurement and Interpretation of the Ankle-Brachial Index: A Scientific Statement From the American Heart Association. *Circulation* **2012**, *126*, 2890–2909. https://doi.org/10.1161/CIR.0b013e318276fbcb.
58. Johnston, A.L.; Vemulapalli, S.; Gosch, K.L.; Aronow, H.D.; Abbott, J.D.; Patel, M.R.; Smolderen, K.G.; Shishebor, M.; Spertus, J.A.; Jones, W.S. Ankle-Brachial Index in Patients with Intermittent Claudication Is a Poor Indicator of Patient-Centered and Clinician-Based Evaluations of Functional Status. *J. Vasc. Surg.* **2019**, *69*, 906–912. https://doi.org/10.1016/j.jvs.2018.07.039.
59. Itoga, N.K.; Minami, H.R.; Chelvakumar, M.; Pearson, K.; Mell, M.M.; Bendavid, E.; Owens, D.K. Cost-Effectiveness Analysis of Asymptomatic Peripheral Artery Disease Screening with the ABI Test. *Vasc Med* **2018**, *23*, 97–106. https://doi.org/10.1177/1358863X17745371.
60. Lindholt, J.S.; Søgaard, R. Clinical Benefit, Harm, and Cost Effectiveness of Screening Men for Peripheral Artery Disease: A Markov Model Based on the VIVA Trial. *Eur. J. Vasc. Endovasc. Surg.* **2021**, *61*, 971–979. https://doi.org/10.1016/j.ejvs.2021.02.039.
61. McDermott, M.M.; Guralnik, J.M.; Tian, L.; Liu, K.; Ferrucci, L.; Liao, Y.; Sharma, L.; Criqui, M.H. Associations of Borderline and Low Normal Ankle-Brachial Index Values With Functional Decline at 5-Year Follow-Up. *J. Am. Coll. Cardiol.* **2009**, *53*, 1056–1062. https://doi.org/10.1016/j.jacc.2008.09.063.
62. Karnon, J.; Haji Ali Afzali, H. When to Use Discrete Event Simulation (DES) for the Economic Evaluation of Health Technologies? A Review and Critique of the Costs and Benefits of DES. *PharmacoEconomics* **2014**, *32*, 547–558. https://doi.org/10.1007/s40273-014-0147-9.
63. Lindholt, J.S.; Søgaard, R. Population Screening and Intervention for Vascular Disease in Danish Men (VIVA): A Randomised Controlled Trial. *Lancet* **2017**, *390*, 2256–2265. https://doi.org/10.1016/S0140-6736(17)32250-X.
64. Available online: Epidemie-COVID-19-v-Datech-Dopad-Na-Zdravotni-Peci-a-Prevenci.Pdf (accessed on).
65. Hap, K.; Biernat, K.; Konieczny, G. Patients with Diabetes Complicated by Peripheral Artery Disease: The Current State of Knowledge on Physiotherapy Interventions. *J. Diabetes Res.* **2021**, *2021*, 5122494. https://doi.org/10.1155/2021/5122494.
66. Tappenden, P.; Chilcott, J.; Brennan, A.; Squires, H.; Stevenson, M. Whole Disease Modeling to Inform Resource Allocation Decisions in Cancer: A Methodological Framework. *Value Health* **2012**, *15*, 1127–1136. https://doi.org/10.1016/j.jval.2012.07.008.
67. Tappenden, P.; Chilcott, J.; Brennan, A.; Squires, H.; Glynne-Jones, R.; Tappenden, J. Using Whole Disease Modeling to Inform Resource Allocation Decisions: Economic Evaluation of a Clinical Guideline for Colorectal Cancer Using a Single Model. *Value Health* **2013**, *16*, 542–553. https://doi.org/10.1016/j.jval.2013.02.012.
68. Lord, J.; Willis, S.; Eatock, J.; Tappenden, P.; Trapero-Bertran, M.; Miners, A.; Crossan, C.; Westby, M.; Anagnostou, A.; Taylor, S.; et al. Economic Modelling of Diagnostic and Treatment Pathways in National

Institute for Health and Care Excellence Clinical Guidelines: The Modelling Algorithm Pathways in Guidelines (MAPGuide) Project. *Health Technol. Assess.* **2013**, *17*, 58. https://doi.org/10.3310/hta17580.
69. Corro Ramos, I.; Hoogendoorn, M.; Rutten-van Mölken, M.P.M.H. How to Address Uncertainty in Health Economic Discrete-Event Simulation Models: An Illustration for Chronic Obstructive Pulmonary Disease. Med Decis Making 2020, 40, 619–632, doi:10.1177/0272989X20932145.
70. Stryja, J.; Turoň, J. Wound Healing – Cost-Eff Ectiveness Data from the Providers and Payers Viewpoint. Cesk Slov Neurol N 2017, 80/113, doi:10.14735/amcsnn2017S18.
71. Eddy, D.M.; Hollingworth, W.; Caro, J.J.; Tsevat, J.; McDonald, K.M.; Wong, J.B. Model Transparency and Validation: A Report of the ISPOR-SMDM Modeling Good Research Practices Task Force–7. Med Decis Making 2012, 32, 733–743, doi:10.1177/0272989X12454579.
72. Státní Ústav pro Kontrolu Léčiv. SP-CAU-028 - W: Postup pro Posuzování Analýzy Nákladové Efektivity 2022. Available online: file:///C:/Users/uzivatel/Downloads/05-A-SP-CAU-028-Postup_pro_posuzovani_analyzy_nakladove_efektivity-17012022.pdf
